# Expression analysis of the *Arabidopsis thaliana AtSpen2* gene, and its relationship with other plant genes encoding Spen proteins

**DOI:** 10.1590/1678-4685-GMB-2016-0223

**Published:** 2017-08-28

**Authors:** María Gloria Solís-Guzmán, Gerardo Argüello-Astorga, José López-Bucio, León Francisco Ruiz-Herrera, Joel López-Meza, Lenin Sánchez-Calderón, Yazmín Carreón-Abud, Miguel Martínez-Trujillo

**Affiliations:** 1 Universidad Michoacana de San Nicolás de Hidalgo, Morelia, Michoacán. Mexico; 2 Instituto Potosino de Investigación Científica y Tecnológica, San Luis, S.L.P., Mexico; 3 Universidad Autónoma de Zacatecas. Zacatecas, Zac., Mexico

**Keywords:** Spen, Arabidopsis, vascular bundle, expression

## Abstract

Proteins of the Split ends (Spen) family are characterized by an N-terminal domain, with one or more RNA recognition motifs and a SPOC domain. In *Arabidopsis thaliana*, the Spen protein FPA is involved in the control of flowering time as a component of an autonomous pathway independent of photoperiod. The *A. thaliana* genome encodes another gene for a putative Spen protein at the locus *At4g12640*, herein named *AtSpen2.* Bioinformatics analysis of the AtSPEN2 SPOC domain revealed low sequence similarity with the FPA SPOC domain, which was markedly lower than that found in other Spen proteins from unrelated plant species. To provide experimental information about the function of *AtSpen2, A. thaliana* plants were transformed with gene constructs of its promoter region with *uidA::gfp* reporter genes; the expression was observed in vascular tissues of leaves and roots, as well as in ovules and developing embryos. There was absence of a notable phenotype in knockout and overexpressing lines, suggesting that its function in plants might be specific to certain endogenous or environmental conditions. Our results suggest that the function of *Atspen2* diverged from that of *fpa* due in part to their different transcription expression pattern and divergence of the regulatory SPOC domain.

## Introduction

Several experimental approaches have been used to determine the function of genes in plants: inactivation by disruption (knockout), overexpression using strong or constitutive promoters, single gene or whole transcriptome analysis, and the use of reporter constructs to study expression patterns conferred by regulatory regions. Notwithstanding the variety of existing methods, only approximately 25% of the genes of *Arabidopsis thaliana*, the most-studied plant species, have been functionally characterized (Rhee and Mutwil, 2014). Using different databases, it is possible to determine the presence of potential *cis* elements in the promoters of genes under study, and predict if transcription is developmentally regulated or if it is regulated by specific internal or external factors (Hernández-García and Finer, 2014).

With the growing number of sequenced plant genomes, numerous databases have been established that can be explored by using various bioinformatics tools to predict the functions of genes and their products. From a structural and evolutionary point of view, it has been possible to classify proteins into families, mainly by comparing related sequences and domains and the presence of tertiary structures associated with specific functions that have been preserved during molecular evolution (Martínez, 2011). The current definition of more than 10,000 protein families could be considered, by analogy, as a periodic table for biology, allowing the prediction of protein functions at the molecular level (Sammut *et al.*, 2008). By means of diverse algorithms, protein domains can be predicted from the amino acid sequence to obtain the first clues about molecular function (Marchler-Bauer *et al.*, 2015).

Proteins of the Split ends (Spen) family are characterized by having an N-terminal RRM domain (RNA recognition motif) and a C-terminal conserved SPOC domain (Spen paralogue and orthologue C-terminal) (Kuang *et al.*, 2000). The RRM domain is also known as an RBD (RNA binding domain) or an RNP (Ribonucleoprotein domain); it consists of 90 amino acids and has a structure composed of four beta sheets and two alpha helices that interact with ssDNA and ssRNA. This domain is found in a variety of hnRNPs (heterogeneous nuclear ribonucleoproteins) involved in alternative splicing and in proteins that are components of snRNPs (small nuclear ribonucleoproteins) (Ariyoshi and Schwabe, 2003). The SPOC domain contains approximately 165 amino acid residues and consists of a beta-barrel with seven sheets (β1-β7) framed by six alpha helices (αA-αF) and is involved in protein-protein interactions; this domain has been described among animals, plants and fungi (Sánchez-Pulido *et al.*, 2004).

The Spen proteins of animals are involved in different biological processes. In *Drosophila,* they participate in neuronal cell differentiation, the growth and guidance of axons in the embryo (Chen and Rebay, 2000; Kuang *et al.*, 2000), cell-cycle regulation (Lane *et al.*, 2000), and repression of head identity of the embryonic trunk (Wiellette *et al.*, 1999). Artavanis-Tsakonas *et al.* (1999) stated that the Notch/RBP-Jkappa signaling pathway is involved in several cellular processes during the embryonary development, such as proliferation, differentiation, apoptosis, maintenance of undifferentiated cells, and cell-fate specification. In humans, the Spen SHARP protein (SMRT7/HDAC1-associated repressor protein) participates as a component of this signaling pathway, repressing the transcription of the *Notch* genes by the RBP-Jk-SHARP complex, which recruits a histone deacetylase complex (Oswald *et al.*, 2002). Furthermore, the participation of SHARP in the silencing of the X chromosome in humans has been recently demonstrated: the long non-coding RNA *Xist* associates with SHARP and two other proteins (SAF-A and LBR), forming a complex that activates a histone deacetylase (DHAC3) which is necessary not only for silencing the chromosome but also for the exclusion of the RNA polymerase II (McHugh *et al.*, 2015).

Flowering is a process that entails the change from the vegetative to the reproductive phase in the angiosperms; it depends on the quantitative integration of environmental stimuli with an endogenous development program (Simpson and Dean, 2002). In *A. thaliana* several flowering pathways have been characterized, including the photoperiod-dependent pathway, the gibberellin (GA) pathway, and the autonomous pathway, which is not triggered by light signals but by exposure to low temperatures in combination with some endogenous cues (Simpson, 2004). In the autonomous pathway, the components act regulating the level of the mRNA that encodes the Flowering Locus C (FLC), a transcription factor that repress flowering by antagonizing the activity of some components of the photoperiod-dependent pathway (Hepworth *et al.*, 2002). The *A. thaliana* Spen protein FPA (Flowering Pathway Autonomous) is a key component of the autonomous pathway (Schomburg *et al.*, 2001). It acts in concert with other proteins (FCA and FY) promoting the 3’ end processing and polyadenylation of an antisense *flc* RNA (Hornyik *et al.*, 2010), leading to the transcriptional down-regulation of the sense *flc* RNA, by a mechanism involving the FLD demethylase (Liu *et al.*, 2010). Thus, the flowering time in *A. thaliana* is increased or reduced when the *fpa* gene is either inactivated or overexpressed, respectively (Schomburg *et al.*, 2001).

The crystal structure of the FPA SPOC domain has been determined at high resolution (2.7 Å) and comparisons with homologous domains showed the highest similarity with the SPOC domain of SHARP, hence demonstrating the high conservation of the SPOC structure among far related organisms (Zhang *et al.*, 2016).

In rice (*Oryza sativa*), two genes encoding Spen proteins, *OsRRM* and *OsRRMh,* have been identified. *OsRRM* is expressed in the seed endosperm and the synthesized protein is localized in the nucleus. However, when this gene was silenced by dsRNAi, no differences with the control plants were found in the seeds and whole plant phenotypes, and no change in the flowering time was observed (Chen *et al.*, 2007). The *OsRRMh* gene is expressed in the roots, stem, leaves, and immature seeds, and it is alternatively spliced in different tissues, suggesting its involvement in several biological functions. When this gene was knocked down by dsRNAi, the flowering was retarded and the panicle phenotype was larger, which was in agreement with the down-regulation of two flowering-related genes of rice: *RFT1* and *Hd3a* (Liu and Cai, 2013).

With the sequencing of the *A. thaliana* genome (Arabidopsis Genome Initiative, 2000), it has been possible to identify numerous putative genes, but the functions of many remain enigmatic. In this work, experimental and bioinformatics information was generated and analyzed to unravel the function of the *At4g12640* gene (herein named *AtSpen2*) encoding a putative Spen protein (AtSPEN2). Gene constructs of the *AtSpen2* promoter fused to *uidA::gfp* reporter genes revealed its expression in the vascular tissues of leaves, roots, and embryos, while no apparent changes in growth, development and flowering time were observed under standard growth conditions in *AtSpen2* knockout (KO) mutants or overexpressing (OE) lines compared to WT plants. We discarded that *AtSpen2* has a function related with the flowering time, and suggest that it may have an accessory function in a development process or its participation is conditional and depends on certain endogenous or environmental conditions.

## Materials and Methods

### Similarity analysis of the AtSPEN2 SPOC domain

Sequences were obtained from http://www.ncbi.nlm.nih.gov and http://www.uniprot.org. Multiple sequence alignment was performed with ClustalW. The tree was generated with Mega 6.0 (Tamura *et al.*, 2013), using the Neighbor-Joining method with the proportion of paired differences.

### Construction of a vector to overexpress *AtSpen2*

A region of the *AtSpen2* cDNA was amplified from a plasmid isolated from an *A. thaliana* cDNA Matchmaker^®^ library. The oligonucleotides FOE 5’-AAAAAGCAGGCT GCATGTCATCTAGAGGAAGGGAGAGGATGA-3’ and ROE 5’-AGAAAGCTGGGTGTTAACTCGGTTT AGCTTGTTGAATCTGCTG-3’ were used to generate the *attB1* and *attB2* recombination sites. The amplified DNA fragment was cloned into the pDONR221^®^ (Invitrogen) vector, harboring the sites *attP1* and *attP2*. The recombination reaction was performed using the BP Clonase Enzyme Mix Kit^®^ (Invitrogen), forming the *attL1* and *attL2* recombination sites. The pDONR221^®^ vector with the *AtSpen2* coding region was recombined with the pK2GW7.0 (Ghent University, Belgium) binary vector (harboring the *attR1* and *attR2* recombination sites), using the LR Clonase Enzyme Mix Kit^®^ (Invitrogen). In the final recombinant vector, the strong 35S promoter of the Cauliflower Mosaic Virus (Odell *et al.*, 1985) was joined to the *AtSpen2* coding region.

### Genotyping of the *AtSpen2* knockout mutant (KO)

Seeds of the *A. thaliana* insertional mutant *At4g12640-T-DNA* (KO) were provided by the Salk Institute for Genomic Analysis. Two lines were used: KO-024223 and KO-030932. Lines were genotyped by PCR as previously described (Alonso *et al.*, 2003).

### *Arabidopsis thaliana* genomic DNA extraction and amplification of the promoter region

DNA of the wild-type *A. thaliana* ecotype Col-0 was isolated using the DNAeasy Plant Mini Kit (QIAGEN), following the manufacturer's instructions. The resulting DNA was used to amplify 500 bp of the *AtSpen2* promoter region, using the oligonucleotides: F500 5’-ACATGACGAGCA GATCTACGGAGA-3’ and R500 5’-TCTGCATTCGTC AGATCTATCGCA-3’. The Supermix High Fidelity^®^ PCR kit (Invitrogen) was used following the manufacturer's instructions. The amplification conditions were 95 °C for 5 min, 30 cycles of 95 °C for 1 min, 55 °C for 1 min, 68 °C for 90 s, followed by a final extension at 68 °C for 10 min.

### Construction of a vector to analyze the expression conferred by the *AtSpen2* promoter region

The 500 bp PCR fragment, corresponding to the *AtSpen2* promoter, was cloned into the pCR8^®^/GW/TOPO (Invitrogen) vector, between the *attL1* and *attL2* recombination sites. The sequence and orientation of the cloned fragment was verified by the method of Sanger *et al.* (1977). The resultant vector was recombined with the pKGWFS7 binary vector (Ghent University, Belgium) containing the *attR1* and *attR2* recombination sites. This vector harbors a translational fusion of the *gfp* and *uidA* reporter genes, allowing both to be used for analyzing the expression conferred by the *AtSpen2* promoter region. Recombination was performed using the LR Clonase II enzyme Mix^®^ Kit (Invitrogen).

### *Agrobacterium tumefaciens* and *Arabidopsis thaliana* genetic transformation

The recombined binary vectors were used to transform the *Agrobacterium tumefaciens* strain pGV2260 (McBride and Summerfelt, 1990) by electroporation at 1800 V, using the Eppendorf electroporator 2510. Transformed colonies were selected in LB medium (Luria) with carbenicillin (100 μL/mL), rifampicin (50 μL/mL), spectinomycin (100 μL/mL) and streptomycin (300 μL/mL).

The *A. tumefaciens* strain pGV2260 containing the recombinant binary vector was grown to an absorbance of 0.6 (600 nm), centrifuged for 5 min at 4500 g. The pellet was resuspended in infiltration medium: 0.5X MS (Murashige and Skoog, 1962), 5% sucrose (Bioxon) and 0.05% Silwett L-77. The bacterial suspension was applied to 2–10 mm *A. thaliana* Col-0 inflorescences following the floral DIP method with some modifications (Clough and Bent, 1998; Martínez-Trujillo *et al.*, 2004). Plants were placed in the dark for 12 h and then transferred to light conditions. To collect the seeds, plants were allowed to produce mature siliques. Plant selection was made in 0.2X MS medium, pH 5.7 supplemented with 0.6% sucrose as carbon source and 1% plant agar (Phytotechnology Laboratories A111), with kanamycin (60 μg/mL) for plant selection.

### Expression analysis by real-time quantitative PCR (RT-qPCR)

Total RNA from Col-0, KO- and OE *A. thaliana* lines was isolated from the roots, leaves, and flowers, using Trizol^®^ (Invitrogen). cDNA synthesis was performed using reverse transcriptase H (Thermo Fisher Scientific) and oligo dT. The following oligonucleotides were designed to amplify a 201 bp *AtSpen2* gene fragment: FAt4g 5’-TCCAAAGGGACTCCAGAATG-3’ and RAt4g 5’-CATAACTGCGACCAGGGAAT-3’. A 226 bp fragment of actin was used as the internal reference, using the oligonucleotides FACTINA 5’-TGCCAATCTACGAGG GTTTC-3’, RACTINA 5’-TTCCGATGGAAGAGCT GGT-3’.

### Growth conditions of different *A. thaliana* lines

Seeds of the different *A. thaliana* lines were surface-disinfected by washing with 96% ethanol (v/v in water) for 7 min and then with 20% sodium hypochlorite (v/v in water) for another 7 min. Subsequently, seeds were rinsed five times with sterile deionized water, resuspended in 1 mL, and placed at 4 °C in the dark for 48 h to promote and synchronize germination.

Disinfected seeds were placed in Petri dishes containing 0.2X MS medium, pH 7.0, for plant growth, supplemented with 0.6% sucrose and 1% plant agar. For the selection of genetically transformed plants, kanamycin (60 μg/mL) was added to the medium. Ten days after germination, plants were transferred to pots with a substrate composed of peat moss, perlite, and vermiculite in 3:1:1 ratios at field capacity water. Both Petri dishes and pots were incubated in growth chambers (Percival Scientific AR-95L), under the following conditions: 24 °C, 100 μmol m^2^s^−1^ light intensity, 80% relative humidity, and a 16:8 h light-dark photoperiod.

### Expression analysis of the *uid*A and *gfp* reporter genes

The transgenic plants with the *uidA* reporter gene (Jefferson *et al.*, 1987) were stained with 0.1% 5-bromo-4-cloro-3-indolyl D-glucuronide (x-gluc) in phosphate buffer (NaH_2_PO_4_ and Na_2_HPO_4_, 0.1 M, pH 7), with 4 mM potassium ferrocyanide and potassium ferricyanide for 12 h at 37 °C. A blue compound was generated by conversion of the x-gluc substrate via the activity of the beta-glucuronidase enzyme encoded by *uidA.* Plants were clarified and fixed according to Malamy and Benfey (1997).

The expression of the *gfp* reporter gene was determined by detecting the green fluorescence emitted by the GFP protein (Haseloff and Amos, 1995). To analyze the expression pattern, seedlings were supplied with propidium iodide (1 μg/mL), which emits red fluorescence. Detection of GFP and propidium iodide fluorescence was performed using an Olympus Fluo-View FV1000-PME microscope. To generate the fluorescence of the GFP protein and propidium iodide, excitation wavelengths of 488 nm and 493 nm were used, respectively; photographs were taken at 509 nm and 535 nm wavelengths, respectively.

## Results

### Bioinformatics analysis of the *AtSpen2* gene and the putative AtSPEN2 protein

To provide information about the function of the *AtSpen2* gene, the similarity of the encoded protein with other Spen proteins of plants and particularly of the SPOC domain was performed by a bioinformatics analysis.

**Figure 1:**
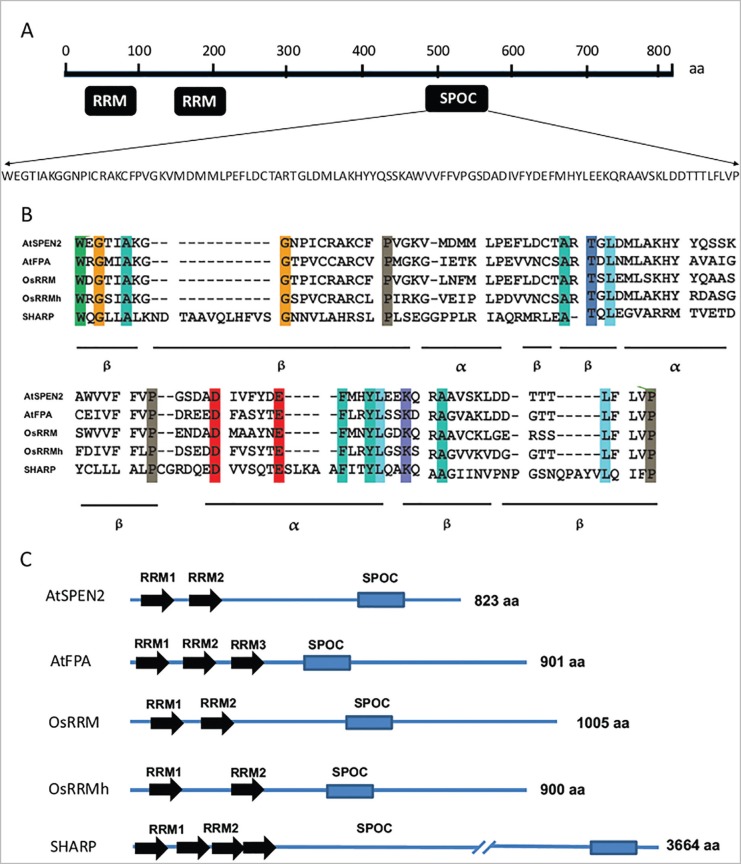
The *AtSpen2* gene encodes a Spen putative protein. (A) The *AtSpen2* gene is located on chromosome 4 from nucleotides 7,462,599 to 7,467,862 in the *A. thaliana* genome (TAIR, The Arabidopsis Information Resource) and encodes an 823 aa putative protein, with two RRM domains (aa 25-89) and (aa 153-223), and a SPOC domain (aa 471-567) (http://www.ncbi.nlm.nih.gov/gene/826877). (B) Alignment of the SPOC sequences in Spen plant and human proteins: AtSPEN2 (*A. thaliana*), AtFPA (*A. thaliana*), OsRRM (*O. sativa*), OsRMMh (*O. sativa*) and SHARP (*Homo sapiens*) is shown. (C) Location of RRM and SPOC domains of Spen proteins: AtSPEN2 (*A. thaliana*), AtFPA (*A. thaliana*), OsRRM (*O. sativa*), OsRMMh (*O. sativa*) and SHARP (*Homo sapiens*).

The *AtSpen2* gene is located on *A. thaliana* chromosome 4 and encodes a putative protein of 823 amino acid (aa) residues. A bioinformatics analysis of this protein (http://www.ncbi.nlm.nih.gov/protein/42566726) predicted two RRM motifs between amino acids 25-90 and 153-223, respectively, and a SPOC domain from aa residues 471-567 (Figure 1A). Hence, it can be concluded that this protein belongs to the Spen family (Sammut *et al.*, 2008). The alignment of the SPOC domain of AtSPEN2 with the homologous domains of other Spen proteins showed the conservation of 18 amino acid residues, and the sequences that participate in the β sheets and α helix structures (Figure 1B). A topological comparison of the RRM and SPOC domains of AtSPEN2 with other described Spen proteins showed that the number of actual or putative RRM domains is either two (AtSpen2, OsRRM and OsRRMh), three (AtFPA) or four (SHARP), but there is only one SPOC domain in the carboxyl side of the protein (Figure 1C).

**Figure 2:**
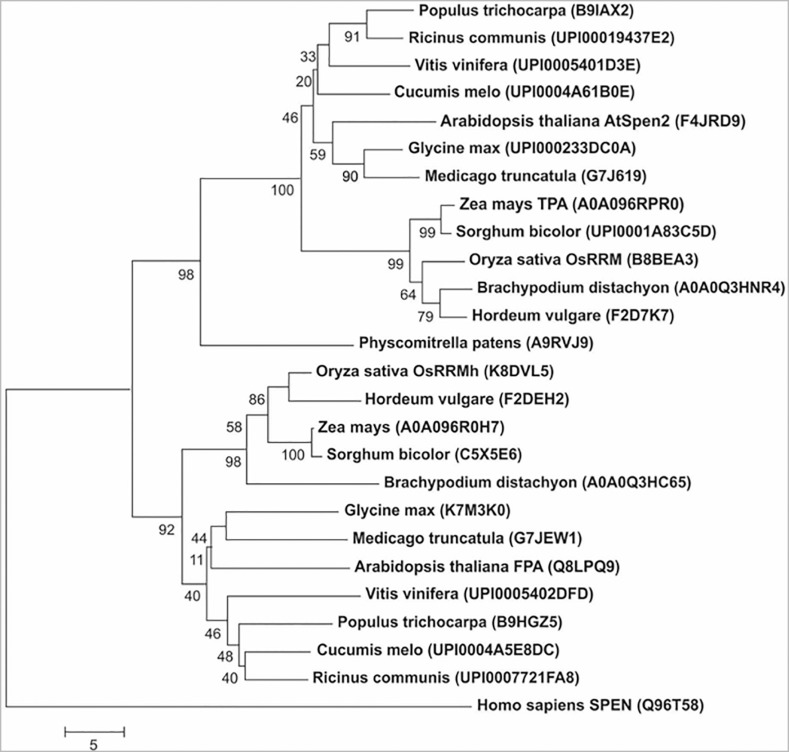
Dendogram inferred using Neighbor-Joining of plant Spen proteins derived from the SPOC domain comparisons. The SPOC sequences of the following species were used: *Arabidopsis thaliana, Populus trichocarpa, Vitis vinifera, Oryza sativa, Hordeum vulgare, Brachypodium distachyon, Physcomitrella patens, Cucumis melo, Medicago truncatula, Shorgum bicolor, Ricinus communis, Glicine max, Zea mays*, and *Homo sapiens* as the external group. Bootstrap values are shown below the branches. The scale is in the units of the number of amino acid differences per sequence, and accession number or locus ID is indicated in parenthesis.

The average size of plant Spen proteins is approximately 900 aa, while the human protein SHARP is almost four times larger, with 3,664 aa. Unlike the RRM domain, which is very diverse (Maris *et al.*, 2005), the SPOC domain is more conserved, allowing evolutionary relationships to be established between proteins. The bioinformatics analysis of SPOC domains in predicted proteins of vascular plants with a sequenced genome revealed that in each plant species there are two genes encoding Spen proteins. Moreover, the SPOC domains of those distinct proteins encoded in the genome of each plant species are grouped in two clearly divergent clades (Figure 2), hence suggesting that the genes encoding Spen proteins of a same clade are orthologous. This also indicates that the *Spen* genes of vascular plants were derived from an antique event of duplication of a single ancestral gene. This supposition is reinforced by the existence of only one Spen gene in the non-vascular plant *Physcomitrella patents*, a moss with a sequenced genome. The moss Spen protein contains a SPOC domain, which displays low sequence identity with both of the major SPOC clades of vascular plants (Figure 2). The comparative analysis of SPOC domains of *A. thaliana* and *Oryza sativa* proteins showed that the domains of AtSPEN2 and OsRRM belong to the same clade, whereas the domains of FPA and OsRRMh, both reportedly involved in flowering time determination in the corresponding plant species, belong to the second major clade (Figure 2).

### Analysis of the *AtSpen2* overexpressing (OE) and knockout (KO) transgenic lines

The binary vector with the *AtSpen2* cDNA fused to the strong 35S promoter of Cauliflower mosaic virus (CaMV) was used to transform *A. thaliana* plants using *A. tumefaciens*. Plants were selected based on kanamycin resistance; homozygous plants were selected. This strategy obtained 13 lines (T3 generation). The *AtSpen2* KO lines were analyzed by PCR and it was found that in KO-024223 the T-DNA was inserted in the exon 3 (nucleotide + 2153), whereas in the KO-030932 line the T-DNA was inserted in the promoter (nucleotide – 413). These KO lines are named KO-E and KO-P in the following sections.

The *in vitro* growth of plants from three OE lines (OE-1, OE-2, OE-3) and two KO lines (KO-E and KO-P), was compared to the WT for 10 days, and then the plants were transferred to pots for the production of flowers and siliques. The following phenotypic variables were analyzed: primary root growth, lateral root number, lateral root density, root biomass, shoot biomass, and flowering time. There was no apparent difference among WT, KO and OE plants in any of the developmental traits analyzed: in Supplementary Figure S1, results of KO-E, OE-1, and WT, are shown as representatives; the other OE lines (OE-2 and OE-3) and the KO-P line, had statistically similar values in the phenotypic variables analyzed. These results discard the participation of the *AtSpen2* gene in the flowering time in the used experimental conditions, differently than its paralogue gene *fpa*.

**Figure 3:**
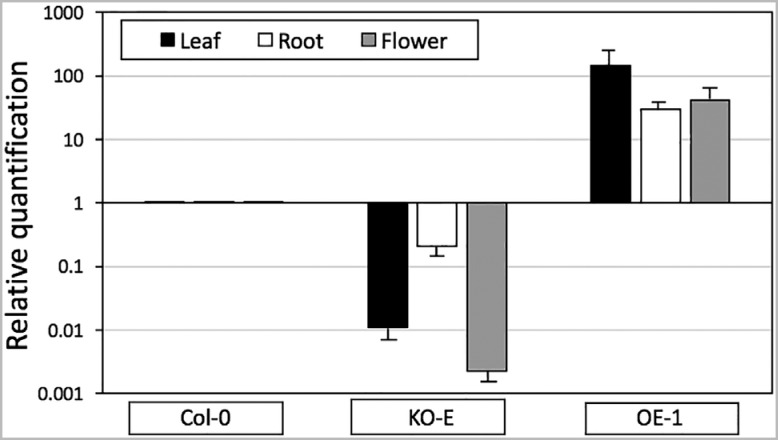
*AtSpen2* expression levels in *A. thaliana* lines. *AtSpen2* gene expression was determined by quantitative PCR, according to the Materials and Methods. Actin was used as the endogenous control. Expression in KO-E and OE-1 lines are reported as fold-change relative to the WT Col-0 line.

The levels of *AtSpen2* transcripts in the OE-1 and KO-E lines used to analyze the phenotypic traits were determined by RT-qPCR. *A. thaliana* Col-0 plants were used as the control, and the expression of the endogenous actin gene was used as the internal reference. In the different organs analyzed, leaf, root, and flower, there was greater expression of the *AtSpen2* gene in the OE-1 line (145.5, 29.5 and 41.2 fold, respectively), and lower in the KO-E line (0.011, 0.21 and 0.0023 fold, respectively), relative to WT seedlings (1, for all organs) (Figure 3). The changes observed in the *AtSpen2* transcript levels in the OE-1 line with respect to the WT plants were more than two magnitude orders in leaves and one magnitude order in root and flowers. In the KO-E line, changes in transcript levels were more variable, with decrements of two magnitude orders in leaves and flowers, but only five-fold in roots. Therefore, we consider that the observed changes in the *AtSpen2* transcript levels in OE-1 and KO-E lines with respect to WT plants are significantly high to be considered typical overexpressing and knockout lines. Furthermore, it is improbable that the KO-E line can produce a normal mRNA, because the exon 3 is interrupted by the T-DNA, as we demonstrated. Although only one endogenous gene was used as control, the experiment was repeated three times with similar results.

### *Cis* elements and expression conferred by the *AtSpen2* promoter to the *uid*A and *gfp* reporter genes

**Figure 4:**
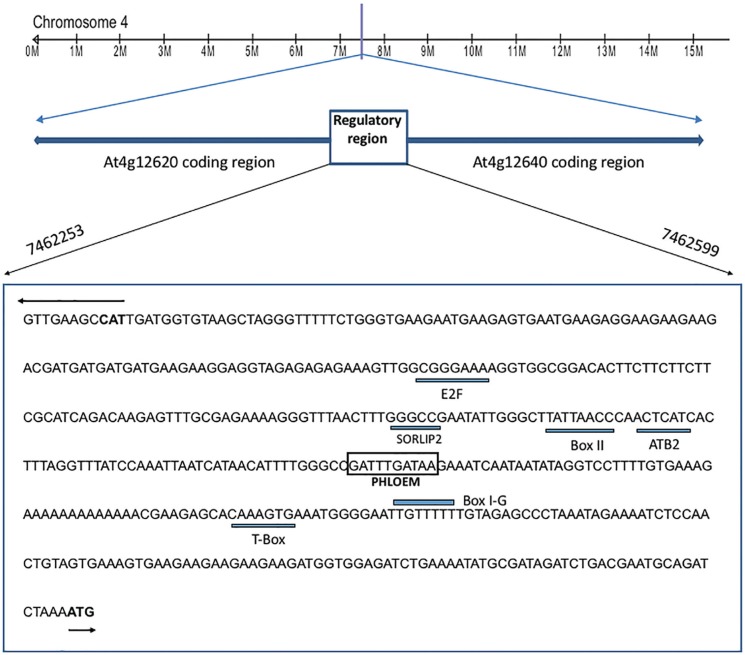
Putative *cis-*regulatory elements in the *AtSpen2* promoter. The nucleotide sequence of 346 bp located 5’ upstream of the *AtSpen2* coding sequence was used for the bioinformatics analysis; this sequence is partially shared with the promoter of the *At4g12620* gene. The underlined sequences correspond to I-Box, GATA-Box and T-Box as part of the *AtSpen2* promoter. The box corresponds to the putative element conferring expression in phloem. Other underlined sequences correspond to *At4g12620 cis* elements: T-Box, ATB2/AtbZIP53/GBF5 BS in ProDH, BoxII, SORLIP2 and E2F.

The promoter region of the *AtSpen2* gene apparently consists of 346 bp, which is the distance that separates it from the nearest gene, *At4g12620*. This promoter lacks a canonical TATA box, which is present in one-third of *A. thaliana* genes at ~ 30 bases upstream of the transcription start site; it also lacks the initiator element (Inr), YYANa/tYY. Some potential *cis*-regulatory elements identified by bioinformatics tools (see Material and Methods) were T-Box (position 7462561-7462566), GATA-Box (position 7462503-7462508), and I-Box (position 7462504-7462509) (AGRIS: Davuluri *et al.*, 2003 and Athena: O'Connor *et al.*, 2005). The GATTTGATA sequence at position 746499-746507 was found, by visual analysis, as a variant of the GAaTTGATA and GAgaTGATA motifs, both reported *cis*-acting elements conferring expression in the phloem (Truernit and Sauer, 1995; Srivastava *et al.*, 2014). This finding is congruent with the tissue-specific expression conferred by the *AtSpen2* promoter, as described in a subsequent section (Figure 4).

The promoter region of *Atspen2* is shared with the *At4g12620* gene, which is located at position 7459646-7462253 (TAIR, The Arabidopsis Information Resource). This gene encodes a protein of the replication origin recognition complex (ORC1B) and its promoter region contains several predicted *cis* elements: T-Box, ATB2/AtbZIP53/GBF5 BS in ProDH, BoxII, SORLIP2 and E2F (AGRIS: Davuluri *et al.*, 2003 and Athena: O'Connor *et al.*, 2005) (Figure 4).

*A. tumefaciens* transformed with a binary vector harboring the *AtSpen2* promoter region fused to the *uidA::gfp* reporter genes was used to transform *A. thaliana* Col-0 plants. The transformed plants were differentiated from untransformed ones by their greenish color and fast growth in media with kanamycin (60 μg/mL). The percentage of plant transformation obtained was 1–1.2%. Plants were selected for homozygosis in the T3 generation. Twelve independent transgenic lines were obtained, 10 of them with detectable expression of the two reporter genes, although with variations in the intensity of expression, possibly due to positional effects of the T-DNA insertion into the genome. The expression analysis of the reporter genes showed that both *uidA* and *gfp* displayed the same expression patterns in the different plant tissues. Accordingly, the results illustrated in the subsequent figures are shown with either one or the other reporter gene, depending on the quality of the images obtained.

**Figure 5:**
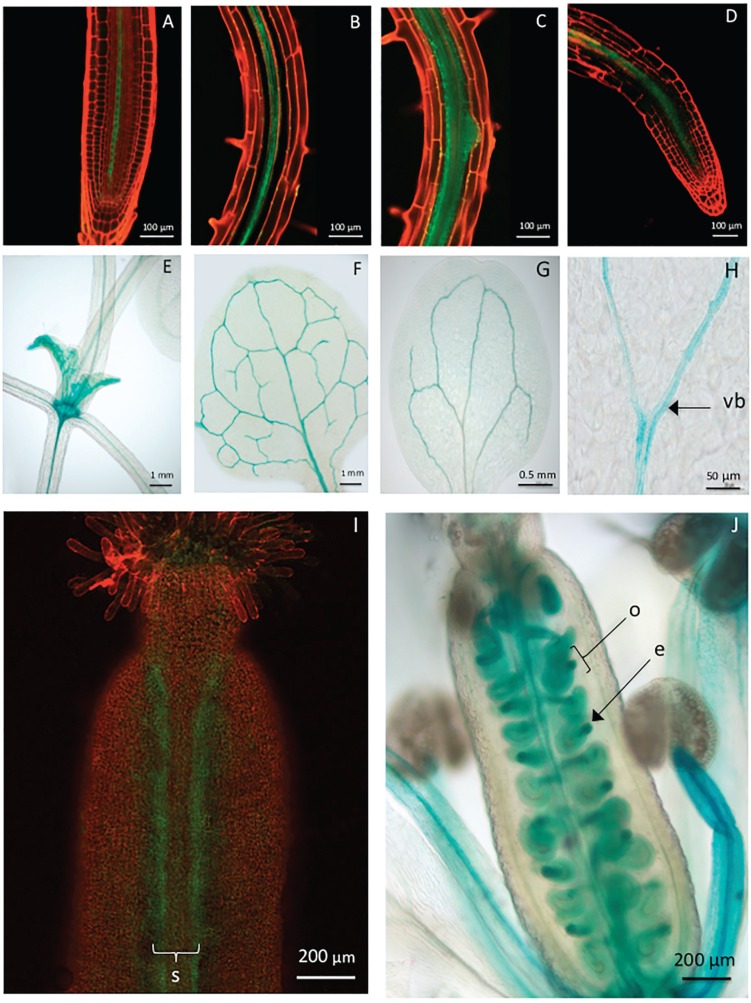
Expression conferred by the *AtSpen2* promoter region in different organs and tissues. (A) Primary root tip; (B) lateral root tip; (C) lateral root primordium; (D) lateral root; (E) stem with forming leaves; (F) true leaf; (G) cotyledonary leaf; (H) vascular bundle; (I) gynoecium in the flower stage 13 (without fertilization); (J) gynoecium in the flower stage 15 (fertilized with developing embryos). In A–D and I, the expression was detected by GFP fluorescence (green). In E-H and J, the expression was detected by the *uidA* reporter gene (blue). Images are representative of 10 replicates. vb, vascular bundle; s, septum; o, ovule; e, embryo.

The expression of the *uidA::gfp* reporter genes was observed in different organs of the plants. In roots, the expression was found in the stele of the differentiated zone, both in the vascular tissue and pericycle. Lateral root primordia originating from pericycle showed expression in all cells, but in the tips of the primary and lateral roots, the expression was detected in the central part of the elongation and meristematic zones, and it was interrupted before reaching the quiescent center. (Figure 5A-D). In the cotyledonary and true leaves, the expression was restricted to the vascular tissue bundles and continued through the petiole (Figure 5E-H).

In the flowers, the expression was observed in the filaments of the stamens, but not in the anthers and pollen grains. Following the stages reported by Smyth *et al.* (1990), in flower stage 12 (flower unopened unfertilized), expression was concentrated in the septum margins (Figure 5I), whereas in the stages 14–15 (fertilized flowers), the expression appeared with greater intensity in the developing embryos (Figure 5J).

## Discussion

In this work, the *AtSpen2* gene of *A. thaliana* and the predicted encoded protein were analyzed by using bioinformatics and experimental methods. The comparative sequence analysis showed that the putative AtSPEN2 protein is different from FPA, the first Spen protein functionally characterized in *A. thaliana*, in both size and overall amino acid sequence: AtSPEN2 exhibits two RRMs in its N-terminal domain whereas FPA displays three RRMs. Systematic comparisons of the AtSPEN2 SPOC domain revealed that it exhibits higher sequence identity to the equivalent domain of Spen proteins encoded by other plant species, including the OsRRM protein of rice (72%), whereas with *A. thaliana* FPA was only 56%. Correspondingly, the SPOC domain of FPA exhibits higher sequence identity to the OsRRMh protein of rice than to AtSPEN2 (Figure 2). These observations indicate that the *AtSpen2* gene is a paralogue of the *fpa* gene of *A. thaliana*, and probably an orthologue of the rice *OsRRM* gene. Thus, it is plausible that the AtSPEN2 protein function has diverged from that of FPA, which is involved in controlling flowering time by the autonomous pathway (Schomburg *et al.*, 2001).

This hypothesis is supported by the results of the phenotypic analysis of *A. thaliana AtSpen2* KO mutants and transgenic OE lines, as flowering time was not modified compared to WT plants. In contrast, inactivation of *fpa* in *A. thaliana* (Schomburg *et al.*, 2001) and its orthologous gene *OsRRMh* in rice (Liu and Cai, 2013) produced a delay in flowering, hence demonstrating that the function of both proteins in flowering control has been preserved through the evolution of angiosperms. It can be hypothesized that the ancestral gene encoding a Spen protein in plants was duplicated before separation of dicots and monocots and that a process of functional divergence took place throughout the subsequent evolution of those plant lineages. The plausible original function of the ancestral protein was the control of flowering, as indicated by the preservation of that function in both FPA and OsRRMh, while the other proteins (AtSPEN2 and OsRRMh) might have one or more functions unrelated to this process.

We functionally analyzed the promoter of *AtSpen2* to establish its pattern of activity in different organs and tissues during plant development, and compared it with the expression conferred by the *fpa* gene promoter (Figure 5, Table 1). This could shed light on potential functional differences between the Spen protein-coding genes examined because promoters have an important role in determining the place (tissue or organ), time, and intensity of transcription, which together with the characteristics of the encoded proteins, largely define the molecular, cellular, and physiological function of these proteins (Hernández-García and Finer, 2014). The absence of a canonical TATA box and an initiator element in the *AtSpen2* promoter indicates that these elements are not the only *cis* elements that are critical for determining transcription initiation of RNA pol II-dependent genes (Narlikar, 2014). Although it has been proposed that yeast and human genes with promoters lacking a TATA box are involved in constitutive processes (Yang *et al.*, 2007), there are numerous exceptions to this rule in those same species and, notably, in plants (Kumari and Ware, 2013). Indeed, the pattern of tissue-specific expression conferred by the *AtSpen2* promoter and its dependence on the development of flower structures suggest that this promoter does not display constitutive transcriptional activity.

**Table 1:** Comparison of organ and tissue-specific expression patterns of the *uidA* reporter gene directed by the *AtSpen2* and *fpa* gene promoters in *Arabidopsis thaliana* (Data of the *fpa* promoter are those reported by Schomburg *et al.*, 2001).

ORGANS/TISSUES	*fpa* EXPRESSION	*AtSpen2* EXPRESSION
COTYLEDONARY LEAVES	Yes **Pattern:** the whole leaf	Yes **Pattern**: vascular tissue
ROOTS	No	Yes **Pattern:** all the stele of primary and lateral roots (vascular tissue and pericycle); lateral root primordia **Time:** all time
MATURE LEAVES	Yes **Pattern:** patches **Time:** only in the two first true leaves	Yes **Pattern:** vascular tissue **Time:** all time
AXILARY MERISTEM INFLORESCENCE	Yes **Pattern:** homogeneous	No
INFLORESCENCE STEM	Yes **Pattern**: homogeneous	Yes **Pattern**: vascular tissue
FLOWER	Yes **Pattern**: not reported **Time:** all time	Yes **Pattern**: septum in stage 12 (non-fertilized flower); developing embryos in stages 14-15. **Time:** onset at stage 12

The most characteristic expression directed by the *AtSpen*2 promoter occurs throughout the vascular tissue (phloem and xylem) and although some *cis* elements have been associated to the specific expression in phloem, the reported sequences are diverse, consisting of 8–13 nucleotides that do not show a significant degree of conservation among distinct promoters. These phloem-specific sequences have been classified into four groups, two of which have been reported in *A. thaliana* (Srivastava *et al.*, 2014). The sequence GATTTGATA found in the *AtSpen2* promoter is very similar to the sequences GAaTTGATA and GAgaTGATA, both reported to confer expression in *Pisum sativum* (Srivastava *et al.*, 2014) and *A. thaliana* (Truernit and Sauer, 1995). Thus, it is possible that this sequence is involved in the phloem expression conferred by the *AtSpen2* promoter. Another possibility is the existence of a different *cis* element (or combination of them) in the *AtSpen2* promoter conferring expression in phloem, but delimiting it needs another approach, such as directed mutagenesis.

Although the reporter gene expression directed by the *AtSpen2* promoter was observed throughout the vascular tissue, it is not clear what function it could have in this tissue, unlike other *A. thaliana* genes that are expressed in the phloem, such as *AHA3* and *SUC2*. The former gene encodes a H^+^-ATPase, which provides energy for SUC2, a sucrose-H^+^ symporter, both contributing to the transport of sucrose in this tissue (Truernit and Sauer, 1995). Another relevant example is the *Cucurbita moschata PP2* gene encoding one of the major proteins involved in the formation of slime plugs in damaged phloem (Guo *et al.*, 2004). While the reported Spen proteins participate in regulating the expression of other genes in animals (Sánchez-Pulido *et al.*, 2004; McHugh *et al.*, 2015) and plants such as FPA, which regulates the expression of the flowering repressor FLC (Hornyik *et al.*, 2010), it is possible that AtSPEN2 contributes to the regulation of a gene whose protein could have a clear function in the phloem.

The root is an organ that allows the plant to be anchored to the substrate and to absorb water and nutrients from the soil and transport them to the aerial parts via the vascular tissue, which is a central stele in *A. thaliana*, both in the primary and lateral roots (Scheres *et al.*, 2002). The proposal that *AtSpen2* function is partly associated with the vascular tissue is reinforced by the expression conferred by the promoter in this tissue, through the root system and the aerial parts, indicating that this gene is being expressed throughout all of the plant vascular system. In the root, the expression of *AtSpen2* extends to the pericycle cell layer, the site where lateral roots originate through robust hormonal and environmental control (Dubrovsky *et al.*, 2008). In transformed *fpa::uidA* plants, expression in the root is not reported (Schomburg *et al.*, 2001, Table1), adding another important difference between *fpa* and *AtSpen2* and reinforcing the notion of a functional difference between these two genes and their encoded proteins. Besides, in our experimental analysis, the expression conferred by the *AtSpen2* promoter in leaves is restricted to the vascular tissue. In contrast, the *fpa* promoter region confers expression mainly in young leaves and in continuous foliar areas that are not associated with the vascular tissue (Schomburg *et al.*, 2001, Table 1), which is an important difference that supports the divergence of the function of both genes in *A. thaliana*.

The expression conferred by the *AtSpen2* promoter in the ovules and developing embryos suggests that this gene plays a role in this stage, but additional studies are required to confirm this because it has been reported that embryogenesis is complex and involves the acquisition of cellular identities and developmental patterns (Capron *et al.*, 2009). Although the *fpa* gene is also expressed in flowers, its pattern is different from that conferred by the *AtSpen2* promoter, because this expression is widely spread throughout the inflorescence and begins in the initial stages of flower development (Schomburg *et al.*, 2001), which is consistent with the function of this gene in the control of flowering time. A comparison of the expression time of the *A. thaliana* and *O. sativa* genes encoding Spen proteins in the developing of the reproductive structures reinforce the hypothesis of divergence of *fpa* and *Atspen2* functions. In the rice genes *OsRRMh* and *OsRRM* (orthologous of *fpa* and *AtSpen2*, respectively), the expression in the former (determined by RT-qPCR) occurs in the leaves and inflorescences (Liu and Cai, 2013), while the *OsRRM* expression (determined by a reporter gene) is associated only with the seed endosperm (Chen *et al.*, 2007). Summarizing this comparison, the expression in the flower of the orthologous genes *fpa* and *OsRRMh* is found at an early flowering stage, in congruence with their reported function in controlling the flowering time, while the orthologous genes *AtSpen2* and *OsRRM* are expressed only in later flower stages.

Although the *AtSpen2* promoter directed reporter gene expression in the transition from the unfertilized flower stage to the flower with developing embryos, we did not observe any changes in the floral development of the plant when the *AtSpen2* gene was inactivated or overexpressed. Some authors have reported that in consequence of gene inactivation there may not necessarily be a morphological change, even if there is a severe physiological alteration; consequently, integral strategies to reveal the function of these genes should be used (Niehaus *et al.*, 2015). The redundancy in the function of genes may account for the absence of a phenotype when a gene is mutated (Bouché and Bouchez, 2001), but in the particular case of *AtSpen2,* this is unlikely because of the following reasons: a) its unique paralogue in *A. thaliana*, the gene *fpa,* has a function in the control of flowering time (Ietswaart *et al.*, 2012), which was not found for *AtSpen2* in this work; b) the SPOC domains of FPA and AtSPEN2 have low sequence identity; and c) the expression conferred by the promoter regions of *AtSpen2* and *fpa* in the organs and tissues of *A. thaliana*, respectively, is completely different. Therefore, it is proposed that the lack of a remarkable phenotype in our KO and OE lines is due to any of the following situations: a) *AtSpen2* is not a master gene in a developmental process, but it may have an accessory function, and b) its participation is conditional and depends on certain endogenous or environmental conditions.

## Conclusions

Taken together, our results demonstrate that the *AtSpen2* gene: a) has a functional promoter that confers expression in the whole vascular tissue, and in the gynoecium and developing embryos, and b) its function in the regulation of the flowering time was discarded, and this is due possibly to the divergence of the transcription expression pattern and low similarity of the SPOC domain with its paralogue *fpa* gene.

## Internet Resources

The databases and bioinformatics tools used in this study are available at the following electronic addresses:

Genetic analysis and molecular biology in *Arabidopsis thaliana*: TAIR The Arabidopsis Information Resource www.arabidopsis.org

Analysis of promoter regions and transcription factors of *Arabidopsis thaliana:* http://arabidopsis.med.ohio-state.edu/

Analysis of nucleotide and protein sequences:

http://www.ncbi.nlm.nih.gov/

http://www.sanger.ac.uk/

http://www.genomesonline.org

Analysis of proteins and domains:

http://pfam.sanger.ac.uk/

http://pfam.janelia.org/

http://pfam.sbc.su.se/

http://www.ncbi.nlm.nih.gov/entrez/query.fcgi?db=cdd

http://www.uniprot.org

http://www.expasy.org/prosite/

## Supplementary material

The following online material is available for this article:

Figure S1 - Analysis of growth and development in *A. thaliana* lines.
