# Supplementary material for: Expression analysis of the Arabidopsis thaliana AtSpen2 gene, and its relationship with other plant genes encoding Spen proteins
Source: Genet Mol Biol. 2017 Aug 28;40(3):643–55. doi: 10.1590/1678-4685-GMB-2016-0223 (PMC5596367; doi:10.1590/1678-4685-GMB-2016-0223)
Supplement: Supplementary file 1 [file 1415-4757-gmb-1678-4685-GMB-2016-0223-Suppl01.pdf]

Genetics and Molecular Biology, 40, 3 (2017)

Supplementary Material to "Expression analysis of the *Arabidopsis thaliana AtSpen2 gene*, and its relationship with other plant genes encoding Spen proteins"

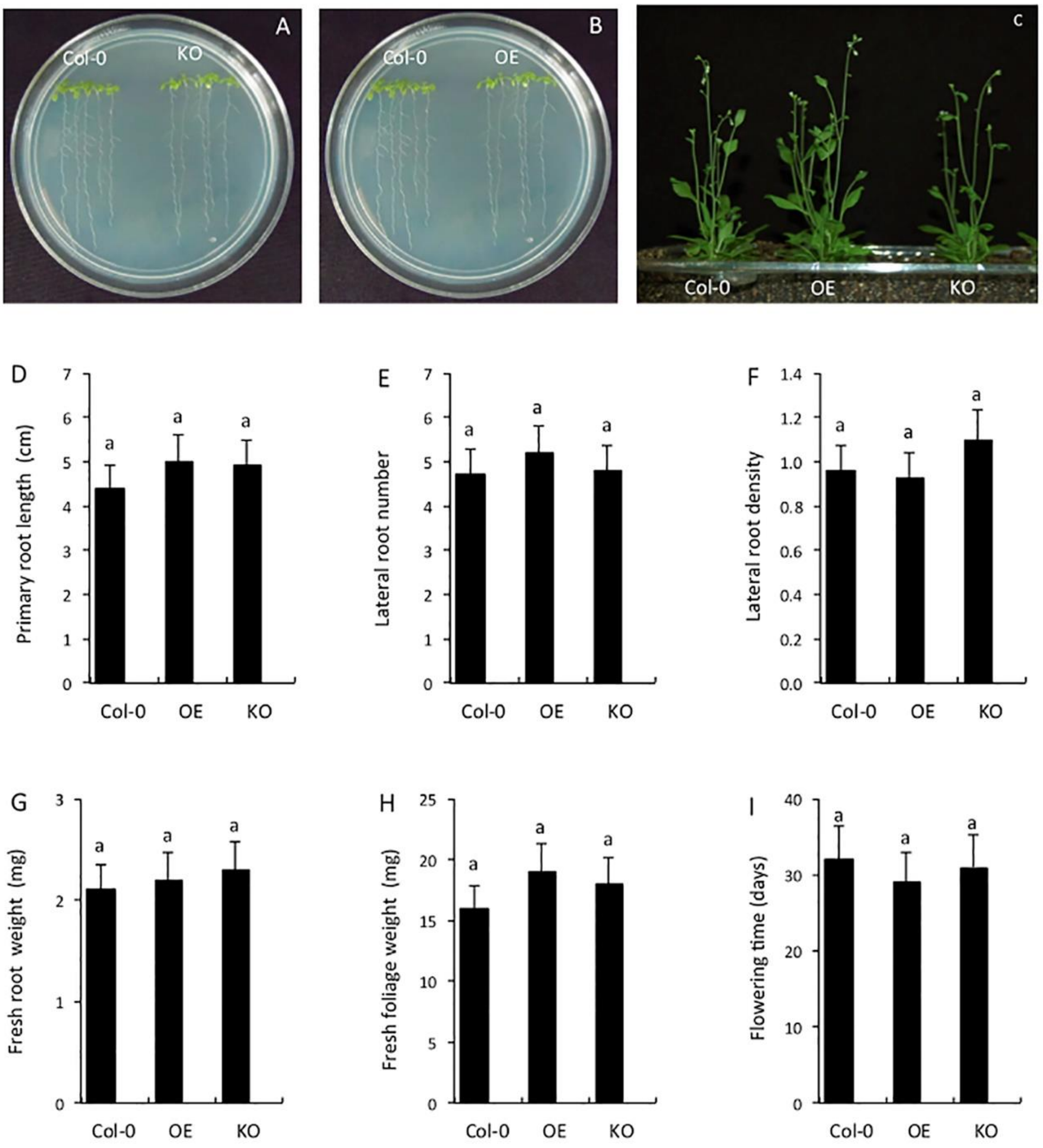

Figure S1 - Analysis of growth and development in *A. thaliana* lines. (A) *In vitro* growth of Col-0 and KO-E lines at 10 days after germination; (B) *in vitro* growth of Col-0 and OE-1 lines at 10 days after germination; (C) growth in pot of Col-0, OE-1 and KO-E lines, at 35 days after germination; (D) primary root growth; (E) number of lateral roots; (F) lateral root density; (G) fresh root weight; (H) fresh foliage weight; (I) flowering time. For D–H, plants were grown *in vitro* and measurements were made 10 days after germination. In I, plants were first grown *in vitro* for 6 days and then transferred to pots. Bars indicate the confidence interval for $\alpha = 0.05$. Different letters indicate significance with the Tukey's test ($p \leq 0.05$, $n = 15$).
